# A complete chloroplast genome of bamboo cultivar *Phyllostachys edulis* f. *bicolor* (Poaceae: Bambusoideae)

**DOI:** 10.1080/23802359.2023.2204168

**Published:** 2023-04-24

**Authors:** Wenxuan Jing, Bo Hu, Rou Wan, Qirong Guo

**Affiliations:** Co-Innovation Center for Sustainable Forestry in Southern China, Nanjing Forestry University, Nanjing, China

**Keywords:** Chloroplast genome, *Phyllostachys edulis* f. *bicolor*, phylogenetic, *Phyllostachys edulis* f. heterocycla

## Abstract

*Phyllostachys edulis* f. *bicolor*, a beautiful ornamental bamboo species, is a new variant of *P. edulis*, with yellow stems and green grooves between nodes. In this study, we assembled and annotated the complete chloroplast (cp) genome of this variety for the first time. The complete cp genome size of *P. edulis* f. *bicolor* was 139,678 bp in length and a total of 130 unique genes were annotated, including 85 protein-coding genes, 37 tRNA encoding genes, and eight rRNA encoding genes. Phylogenetic analysis results provided evidence that *P. edulis* f. *bicolor* was closely related to *P. edulis* ‘heterocycla’. This study contributes to better understanding of intraspecific type evolution of *P. edulis*.

## Introduction

*Phyllostachys edulis* f. *bicolor* (*Phyllostachys edulis* (Carr.) H. de Lehaie f. *bicolor* (Nakai) G. H. Lai 2012) is a new variant of *Phyllostachys* showing color variation, and considered an excellent ornamental plant (Chen 2013). In contrast to the archetype, its bamboo culm is mainly yellow, but the longitudinal groove on the side of the internode branches is green, with a few thin green longitudinal stripes outside the groove. Some leaves are green with yellowish thin longitudinal stripes, and the colors of the bamboo sheath, culm sheath, and patches are lighter. The varieties are mainly distributed in south of the Huaihe River and north of Nanling (Lai 2012; Bu and Zhang 2015; Zeng et al. 2018). In this study, using high-throughput sequencing technology, a complete chloroplast (cp) genome of *P. edulis* f. *bicolor* was obtained for the first time to provide a reference for understanding the evolution of intraspecific types of *P. edulis*.

## Materials

**Figure 1.**
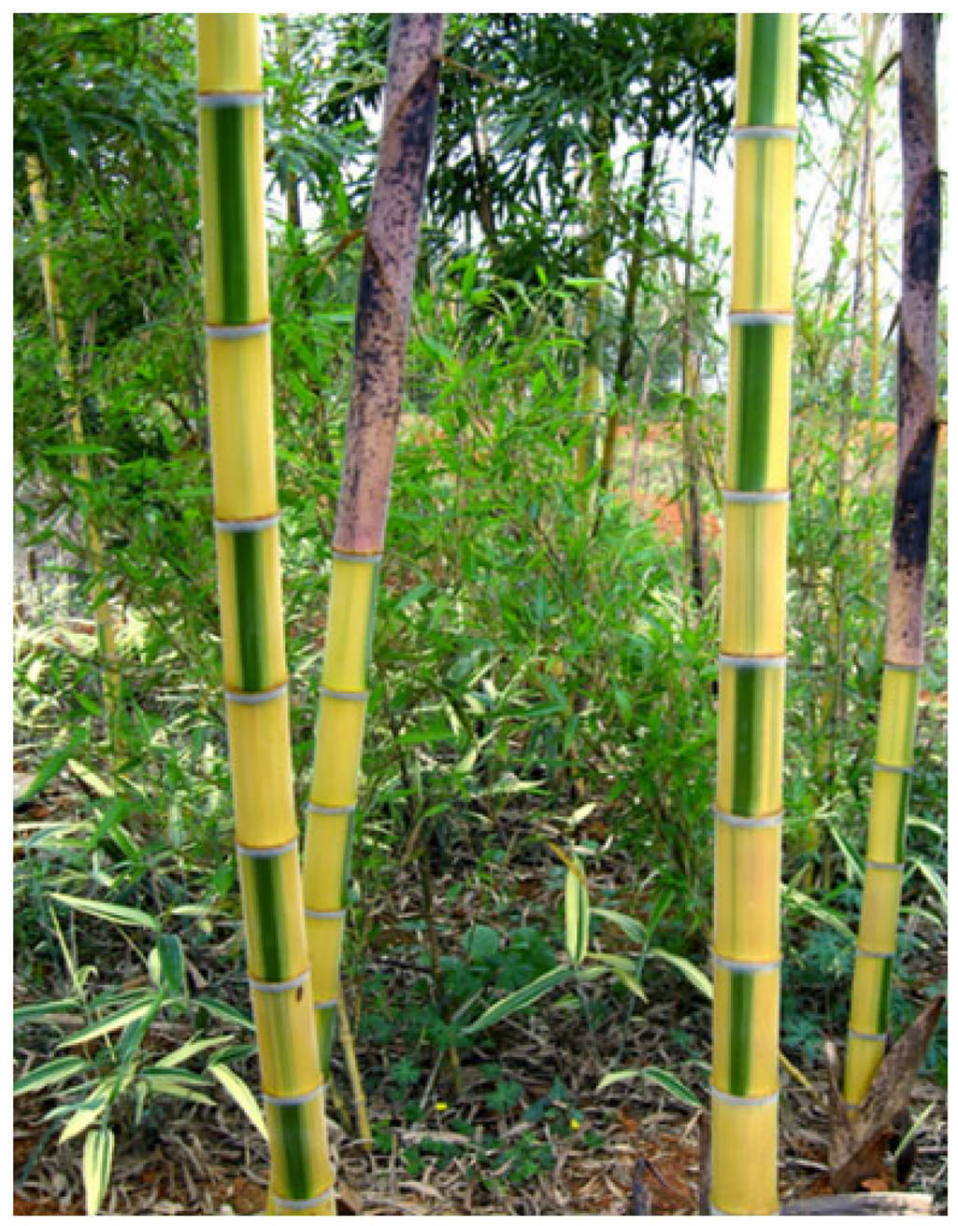
*Phyllostachys edulis* f. *bicolor.* The main performance is that the bamboo culm is yellow, and the groove part is green; some leaves have yellow and green stripes. The photos of *P. edulis f. bicolor* were taken by the authors in Anji county, Zhejiang Province, China.

Samples were collected from an experimental bamboo forest (Figure 1) at Nanjing Forestry University (118.817139° E, 32.078625° N). Voucher specimens of *P. edulis* f. *bicolor* (collector: Qirong Guo, qrguo@njfu.edu.cn, voucher number NJFU-G2017P11) were deposited in the laboratory of the herbarium at the Co-Innovation Center for Sustainable Forestry in Southern China, Nanjing Forestry University.

## Methods

Genomic DNA was extracted using a Qiagen Plant Genomic DNA Prep Kit (Sangon Biotech, Shanghai, China), sequenced on the Illumina Hiseq 2500 platform, and deposited to NCBI under the record number SRR18455992. Data quality control was performed using fastp (Chen et al. 2018), and Novoplasty (v4.0) was used to assemble high-quality paired data into a complete cp genome. Genome annotation was performed using GeSeq (Tillich et al. 2017). Alignment was performed using the *P. edulis* cp genome sequence combined with manual manipulation, using Geneious R8 (Biomatters Ltd, Auckland, New Zealand) for correction. tRNA genes were further validated using the tRNAscan-SE online web server (Schattner et al. 2005). A gene map of the annotated *P. edulis* f. *bicolor* cp genome was drawn online using CPGview (Liu et al. 2023), and the annotated *P. edulis* f. *bicolor* cp genome sequence was submitted to NCBI (accession number: OM084949). To confirm the phylogenetic position of *P. edulis* f. *bicolor*, we obtained 11 published *Phyllostachys* varieties cp genomes from NCBI, and four *Phyllostachys* varieties cp genomes from the China National GeneBank DataBase (CNGBdb). The phylogeny was inferred using the maximum-likelihood (ML) method. MAFFT software was used for sequence alignment (Katoh and Standley 2013), and the phylogenetic tree was constructed using IQ-TREE (Minh et al. 2020). The best-fitting model was selected by ModelFinder (Kalyaanamoorthy et al. 2017) and implemented in IQ-TREE.

## Results

### Analysis of chloroplast genome characteristics

**Figure 2.**
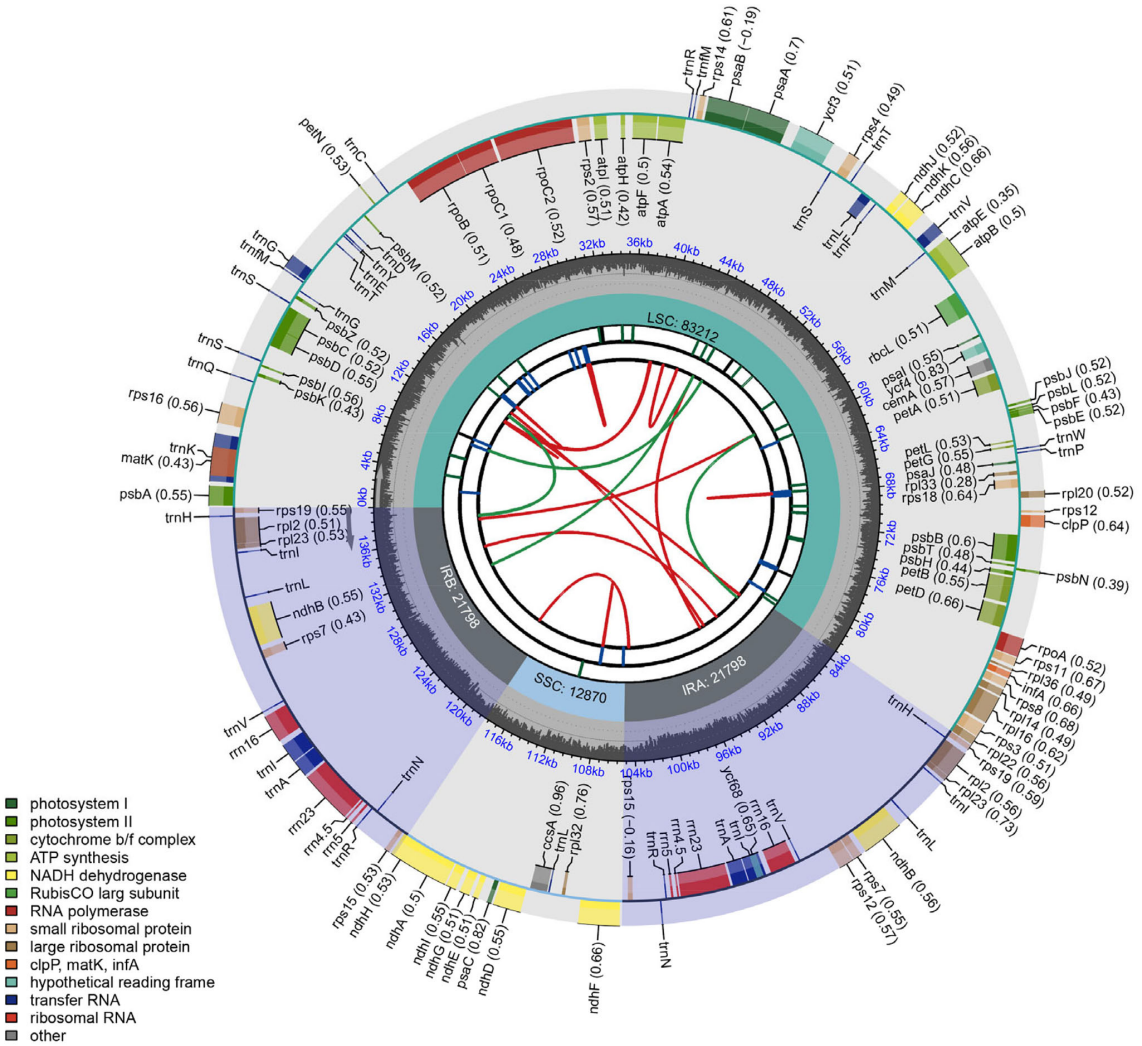
Schematic map of overall features of the chloroplast genome of *Phyllostachys edulis* f. *bicolor.*

The full length of the *P. edulis* f. *bicolor* cp genome (Figure 2) of 139,678 bp is comprised of a large single-copy region (LSC, 83,212 bp), small single-copy region (SSC with 12,870 bp), and two inverted repeat regions (IR with 21,798 bp). The GC content of the total genome was 38.9%, and the GC content of the LSC, SSC, and IR regions was 37.0%, 33.2%, and 44.2%, respectively. There were 130 unique genes, including 85 protein-coding genes, 37 tRNA encoding genes, and eight rRNA encoding genes. According to their function, all annotated genes were divided into four main categories: genes related to photosynthesis (*n* = 45), genes related to self-replication (*n* = 75), genes encoding other proteins (*n* = 5), and genes of unknown function (*n* = 5), with 19 of them (*ndhB*, *rps7*, *rps15*, *rps19*, *rpl2*, *rpl23*, *ycf2*, *trnA-UGC*, *trnH-GUG*, *trnI-CAU*, *trnI-GAU*, *trnL-CAA*, *trnN-GUU*, *trnR-ACG*, *trnV-GAC*, *rrn4.5*, *rrn5*, *rrn16*, and *rrn23*) being double-copy genes.

The map contains six circles. From the center going outward, the first circle shows the forward and reverse repeats connected with red and green arcs, respectively. The second circle and the third circle show tandem repeats and microsatellite sequences marked with short strips, respectively. The fourth circle indicates the position of LSC, SSC, IRA, and IRB, and the fifth circle indicates the GC content. The genes outside the sixth circle are transcribed counterclockwise, while the genes inside are transcribed clockwise. The genes were colored based on their functional categories.

### Phylogenetic analysis of *Phyllostachys edulis* f. *bicolor*

**Figure 3.**
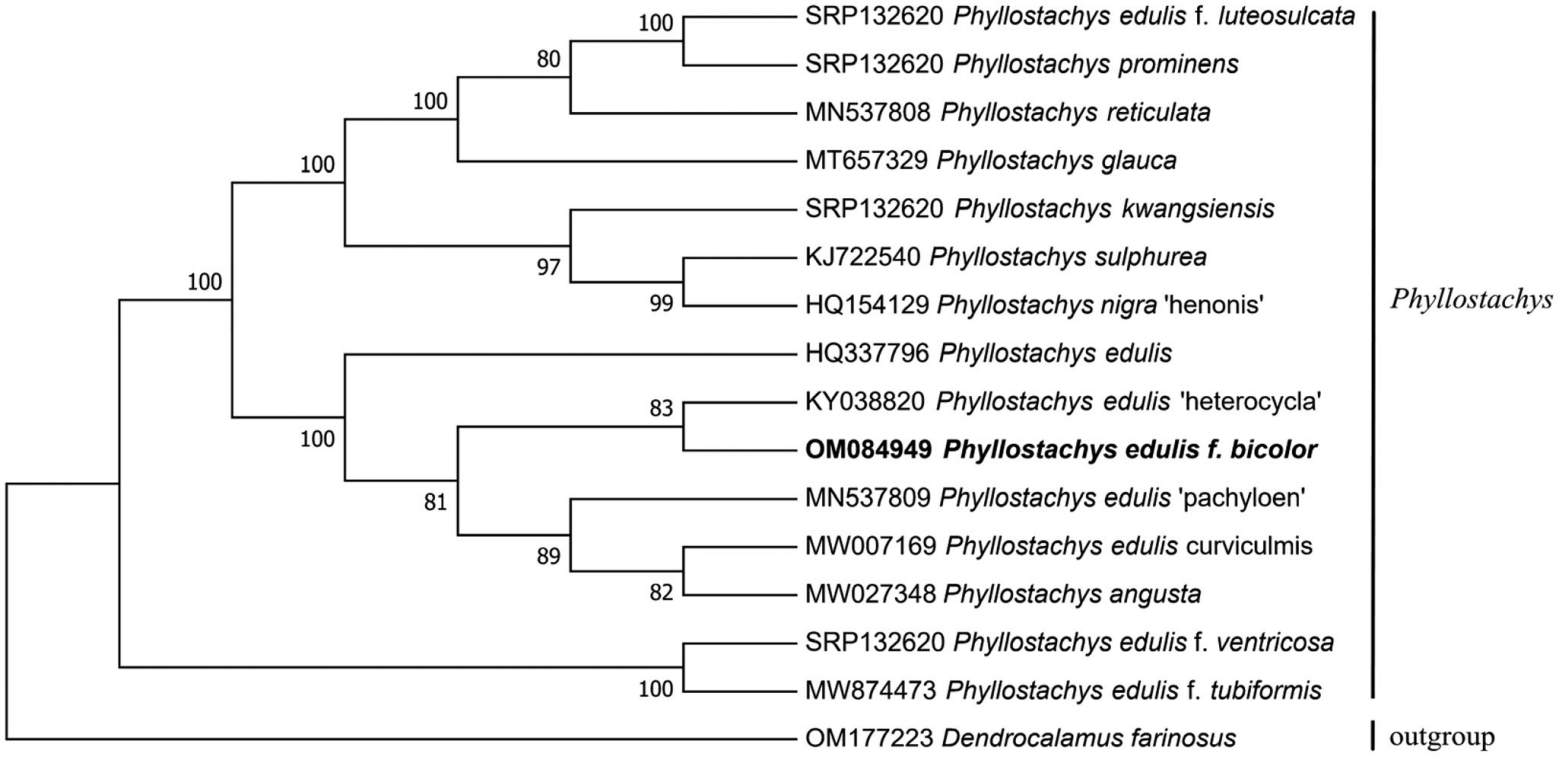
Phylogenetic relationships between *Phyllostachys edulis f. bicolor* and some other species of *Phyllostachys* Varieties. Bootstrap support values are given at the nodes.

The phylogenetic relationships of some *P. edulis* varieties are described using phylogenetic analysis. *P. edulis* ‘heterocycla’, *P. edulis* f. *bicolor*, *P. edulis* ‘pachyloen’, *P. edulis curviculmis*, and *P. angusta* formed a small branch, with *P. edulis. P. edulis* and *P. angusta* each gathered into one branch, *P. edulis* f. *bicolor* and *P. edulis* ‘heterocycla’ clustered together, and *P. edulis curviculmis* and *P. angusta* forming another cluster. This indicated that they were closely related to each other and had notable collinearity in the evolution of intraspecific types of *Phyllostachys* (Figure 3).

The phylogenetic tree is constructed by the ML method. The sequences used for tree construction are as follows: *P. edulis* f. *luteosulcata* (SRP132620)*. P. prominens* (SRP132620)*. P. kwangsiensis* (SRP132620)*. P. edulis f. ventricose* (SRP132620)*. P. reticulata* (MN537808; Huang et al. 2019). *P. glauca* (MT657329; Cao et al. 2020)*. P. sulphurea* (KJ722540; Gao and Gao 2016)*. P. nigra* ‘henonis’ (HQ154129)*. P. edulis* (HQ337796)*. P. edulis* ‘heterocycla’ (KY038820; Dan et al. 2017)*. P. edulis f. bicolor* (OM084949). *P. edulis* ‘pachyloen’ (MN537809; Huang et al. 2019). *P. edulis curviculmis* (MW007169; Gao et al. 2021)*. P. angusta* (MW027348; Yu et al. 2021)*. P. edulis f. tubiformis* (MW874473; Liu et al. 2022)*. Dendrocalamus farinosus* (OM177223; Pei et al. 2022).

## Discussion and conclusions

China’s *Phyllostachys* resources are rich; there are several varieties with significant phenotypic genetic difference among them (Zhang et al. 2007). In this study, we successfully assembled the complete cp genome of *Phyllostachys edulis* f. *bicolor* for the first time and selected the complete cp genome data of 15 other *Phyllostachys* varieties for phylogenetic analysis. The results showed that *P. edulis* f. *bicolor* was closely related to *P. edulis* ‘heterocycla’. The findings were consistent with those of previous studies on *P. edulis curviculmis* (Gao et al. 2021), where *P. edulis* f. *bicolor* formed a small branch with *P. edulis curviculmis* and *P. edulis* ‘pachyloen’, all of which are members of the *Phyllostachys* genus. Moreover, the cp genome size and structure of *P. edulis* f. *bicolor* and *P. edulis curviculmis* were the same, which further indicated the genetic relationship between the two. Elucidation of the complete cp genome of *P. edulis* f. *bicolor* provides important data for the phylogeny of *Phyllostachys* varieties that will help us in better understanding of the evolution of intraspecific types of *Phyllostachys*.

## Supplementary Material

Supplemental Material

## Data Availability

The genome sequence data that support the findings of this study are openly available in GenBank of NCBI at https://www.ncbi.nlm.nih.gov, under the accession number OM084949. The associated BioProject, SRA, and Bio-Sample numbers are PRJNA818118, SRR18455992, and SAMN26816507, respectively.

## References

[CIT0001] Bu LQ, Zhang XM. 2015. Ornamental features of *Phyllostachys* spp. and its landscaping design. World Bamboo Rattan. 13(4):33–36.

[CIT0002] Cao B, Ge TT, Ding SX, Guo CC. 2020. The complete chloroplast genome of *Phyllostachys glauca* (Bambusoideae), a dominant bamboo species in limestone mountains endemic to China. Mitochondrial DNA B Resour. 5(3):3193–3194. 33458108 10.1080/23802359.2020.1810152 PMC7783095

[CIT0003] Chen S, Zhou Y, Chen Y, Gu J. 2018. Fastp: an ultra-fast all-in-one FASTQ preprocessor. Bioinformatics. 34(17):i884–i890. 30423086 10.1093/bioinformatics/bty560 PMC6129281

[CIT0004] Chen TG. 2013. *Phyllostachys edulis* f. *bicolor*. World Bamboo Rattan. 11(2):12–13.

[CIT0005] Dan Z, Yuan L, Li ZG. 2017. The complete chloroplast genome sequence of *Phyllostachys heterocycla*, a fast-growing non-timber bamboo (Poaceae: Bambusoideae). Conserv Genet Resour. 9(2):217–219.

[CIT0006] Gao J, Gao LZ. 2016. The complete chloroplast genome sequence of the *Phyllostachys sulphurea* (Poaceae: Bambusoideae). Mitochondrial DNA A DNA Mapp Seq Anal. 27(2):983–985. 24938113 10.3109/19401736.2014.926516

[CIT0007] Gao LQ, Li YL, Zhang WG, Yang GY. 2021. The complete chloroplast genome of *Phyllostachys edulis curviculmis* (Bambusoideae): a newly ornamental bamboo endemic to China. Mitochondrial DNA B Resour. 6(3):941–942. 33796691 10.1080/23802359.2021.1888663 PMC7971251

[CIT0008] Huang NJ, Li JP, Yang GY, Yu F. 2019. Two plastomes of *Phyllostachys* and reconstruction of phylogenic relationship amongst selected *Phyllostachys* species using genome skimming. Mitochondrial DNA B Resour. 5(1):69–70. 33366426 10.1080/23802359.2019.1696244 PMC7721041

[CIT0009] Kalyaanamoorthy S, Minh B, Wong TKF, Von Haeseler A, Jermiin LS. 2017. ModelFinder: fast model selection for accurate phylogenetic estimates. Nat Methods. 14(6):587–589. 28481363 10.1038/nmeth.4285 PMC5453245

[CIT0010] Katoh K, Standley DM. 2013. MAFFT multiple sequence alignment software version 7: improvements in performance and usability. Mol Biol Evol. 30(4):772–780. 23329690 10.1093/molbev/mst010 PMC3603318

[CIT0011] Lai GH. 2012. A reconsideration of taxonomic position of some infraspecific mutants in the genus *Phyllostachys* (Bambusoideae). J Anhui Agric Sci. 40(8):4621–4625.

[CIT0012] Liu S, Ni Y, Li J, Zhang X, Yang H, Chen H, Liu C. 2023. CPGview: a package for visualizing detailed chloroplast genome structures. Mol Ecol Resour. 23(3):694–704. 36587992 10.1111/1755-0998.13729

[CIT0013] Liu XM, Liu L, Li LB, Yue JJ. 2022. The complete chloroplast genome of *Phyllostachys edulis* f. *tubiformis* (Bambusoideae): a highly appreciated type of ornamental bamboo in China. Mitochondrial DNA B Resour. 7(1):185–187. 35028414 10.1080/23802359.2021.2018945 PMC8751489

[CIT0014] Minh BQ, Schmidt HA, Chernomor O, Schrempf D, Woodhams MD, Von Haeseler A, Lanfear R. 2020. IQ-TREE 2: new models and efficient methods for phylogenetic inference in the genomic era. Mol Biol Evol. 37(5):1530–1534. 32011700 10.1093/molbev/msaa015 PMC7182206

[CIT0015] Pei JL, Wang Y, Zhuo J, Gao HB, Vasupalli N, Hou D, Lin XC. 2022. Complete chloroplast genome features of *Dendrocalamus farinosus* and its comparison and evolutionary analysis with other Bambusoideae species. Genes. 13(9):1519–1533. 36140690 10.3390/genes13091519 PMC9498922

[CIT0016] Schattner P, Brooks AN, Lowe TM. 2005. The tRNAscan-SE, snoscan and snoGPS web servers for the detection of tRNAs and snoRNAs. Nucleic Acids Res. 33(Web Server):W686–W689. 15980563 10.1093/nar/gki366 PMC1160127

[CIT0017] Tillich M, Lehwark P, Pellizzer T, Ulbricht-Jones ES, Fischer A, Bock R, Greiner S. 2017. GeSeq- versatile and accurate annotation of organelle genomes. Nucleic Acids Res. 45(W1):W6–W11. 28486635 10.1093/nar/gkx391 PMC5570176

[CIT0018] Yu Z, Lin YZ, Tao W, Guo FC, Yi FH, Jin JY. 2021. The complete chloroplast genome of *Phyllostachys angusta* McClure (Poaceae). Mitochondrial DNA B Resour. 6(1):187–188. 33537438 10.1080/23802359.2020.1860703 PMC7832504

[CIT0019] Zeng QN, Yu L, Cheng P, Wang HX, Peng JS. 2018. A study of culm color characteristic and type classification of *Phyllostachys edulis* cv. Tao Kiang. World Bamboo Rattan. 16(4):32–35.

[CIT0020] Zhang SF, Ma QX, Ding YL. 2007. Genetic diversity analysis of moso bamboo based on morphological characterization. J Bamboo Res. 7(3):16–21.

