# Supplementary material for: A complete chloroplast genome of bamboo cultivar Phyllostachys edulis f. bicolor (Poaceae: Bambusoideae)
Source: Mitochondrial DNA B Resour. 2023 Apr 24;8(4):532–5. doi: 10.1080/23802359.2023.2204168 (PMC10132244; doi:10.1080/23802359.2023.2204168)
Supplement: Supplemental Material [file TMDN_A_2204168_SM7461.docx]

**Supplementary materials：**

**Fig S1.** The coverage figure of the complete chloroplast genome of *Phyllostachys edulis* f. *bicolor*.

**Fig S2.** Schematic map of the cis and trans splicing genes in the chloroplast genome of *Phyllostachys edulis* f. *bicolor*.


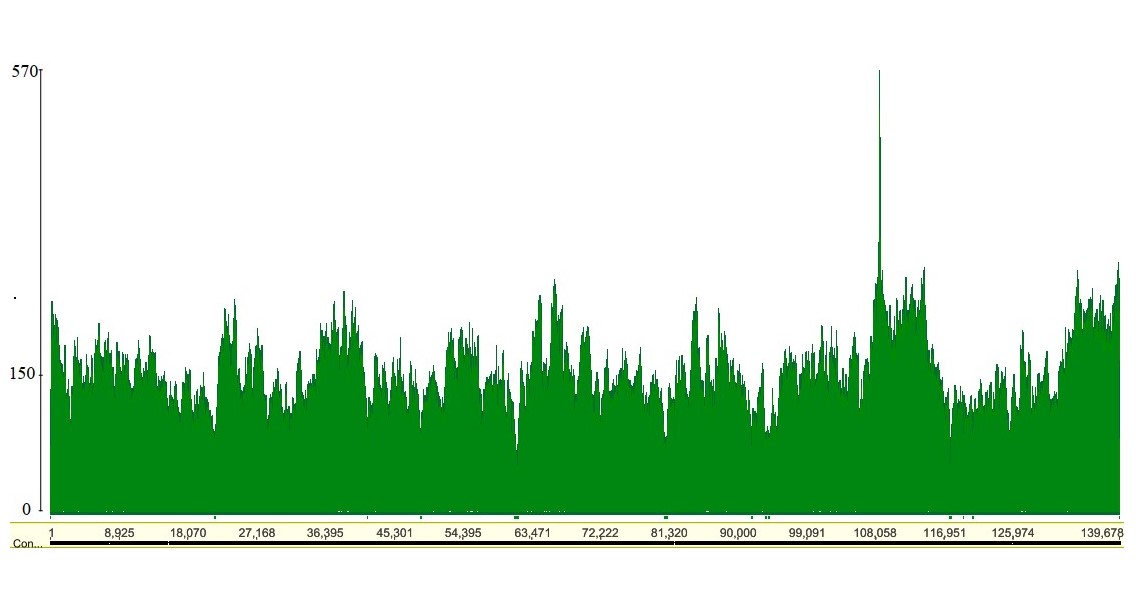


**Fig S1.** The coverage figure of the complete chloroplast genome of *Phyllostachys edulis* f. *bicolor*. This diagram is generated using Bowtie software by comparing the sequencing data with the assembled sequence, and then visualizing it through Geneious. The height of the green graph indicates the number of sequences at each location. It can be used as evidence for chloroplast genome assembly of *P. edulis* f. *bicolor.*


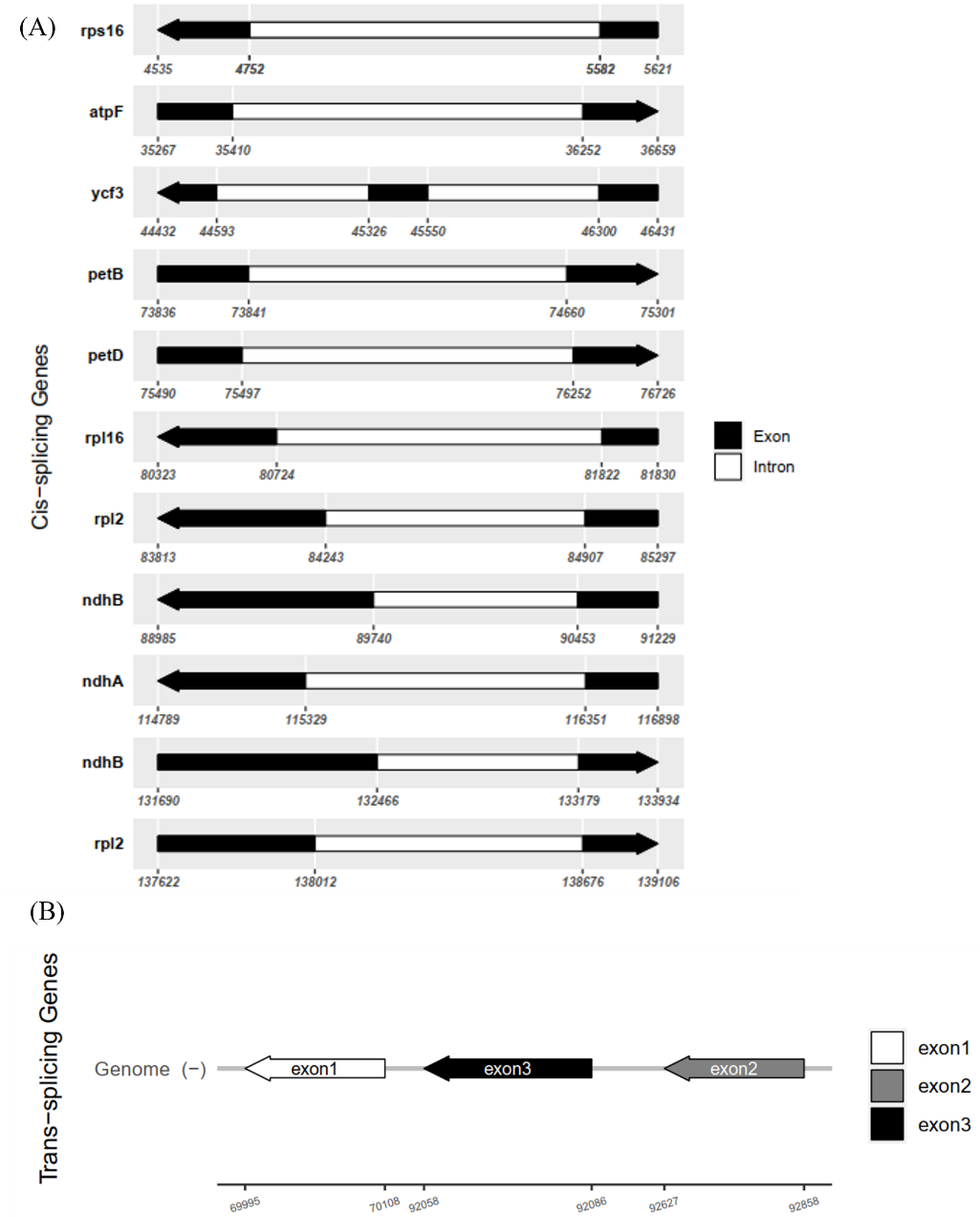


**Fig S2.** Schematic map of the cis-splicing genes (A) and trans-splicing gene rps12 (B) in the chloroplast genome of *Phyllostachys edulis* f. *bicolor*. The exons are shown in black; the introns are shown in white. The arrow indicates the sense direction of the gene. The map was generated using CPGview.
